# Primary diffuse large B-cell lymphoma of the breast with rare growth pattern mimics lobular carcinoma: a case report

**DOI:** 10.3389/fonc.2025.1646986

**Published:** 2025-10-13

**Authors:** Si Su, Jian Sun, Boju Pan, Congwei Jia

**Affiliations:** Department of Pathology, Peking Union Medical College Hospital, Chinese Academy of Medical Sciences & Peking Union Medical College, Beijing, China

**Keywords:** DLBCL, breast, primary, histology, LCA

## Abstract

We report a case of a 34-year-old female presenting with a rapidly enlarging right breast mass. Ultrasonographic evaluation revealed a large solid mass accompanied by ipsilateral axillary lymphadenopathy. Histopathological examination of the biopsy specimen showed an unusual rowth pattern of atypical cells, characterized by perivascular “target-like” and “soldier-like” arrangements, which closely mimicked lobular carcinoma and posed significant challenges to the diagnostic process. Immunohistochemical (IHC) staining was crucial in differentiating this lymphoma from breast carcinoma, with positive results for LCA, CD20 and CD19, and negative for ER and PR. Following multiple rounds of IHC testing, the final diagnosis was confirmed as diffuse large B-cell lymphoma (DLBCL), non-special type (NOS). Further staging revealed no distant metastasis. The case of primary breast DLBCL highlights us a rare perivascular target-like growth pattern of tumor cells. The inclusion of LCA in the IHC panel is recommended to aid in accurate diagnosis and guide appropriate treatment, especially when histological patterns are ambiguous.

## Introduction

Diffuse large B-cell lymphoma (DLBCL) is the most prevalent subtype of non-Hodgkin’s lymphomas (NHLs) worldwide (1, 2). However, primary breast lymphoma (PBL) is extremely rare, accounting for only 0.13% of all malignant breast tumors and approximately 1% of all NHL cases (3–5). Therefore, primary DLBCL of the breast is exceptionally uncommon. PBL was first defined by Wiseman and Liao in 1972 with strict criteria, which is a lymphomatous infiltration in the breast or close to mammary tissue in anatomic proximity, with extension limited to the ipsilateral axillary lymph nodes (6). It is an aggressive disease that exerts a profound adverse impact on patient prognosis. According to literature, the 5-year overall survival rates for patients with indolent and aggressive PBL are 75% and 54%, respectively (7). The standard first-line treatment for DLBCL is typically 4–6 cycles of the R-CHOP regimen (rituximab, cyclophosphamide, doxorubicin, vincristine, and prednisone).

However, it is difficult to distinguish between breast cancer and breast lymphoma only based on clinical symptoms and imaging data, because of similar painless and palpable masses. The diagnosis relies heavily on histopathological examination of biopsy specimens, combined with immunohistochemical testing (8). In this report, we present a rare case of primary DLBCL of the breast with an unusual growth pattern that closely mimics lobular carcinoma. This distinctive histological feature rendered the diagnostic process for this case particularly challenging and protracted. Through detailed discussion of this case, we aim to enhance the diagnostic accuracy of primary breast lymphoma, thereby ensuring patients receive timely and appropriate treatment to improve their prognosis.

## Case presentation

**Figure 1:**
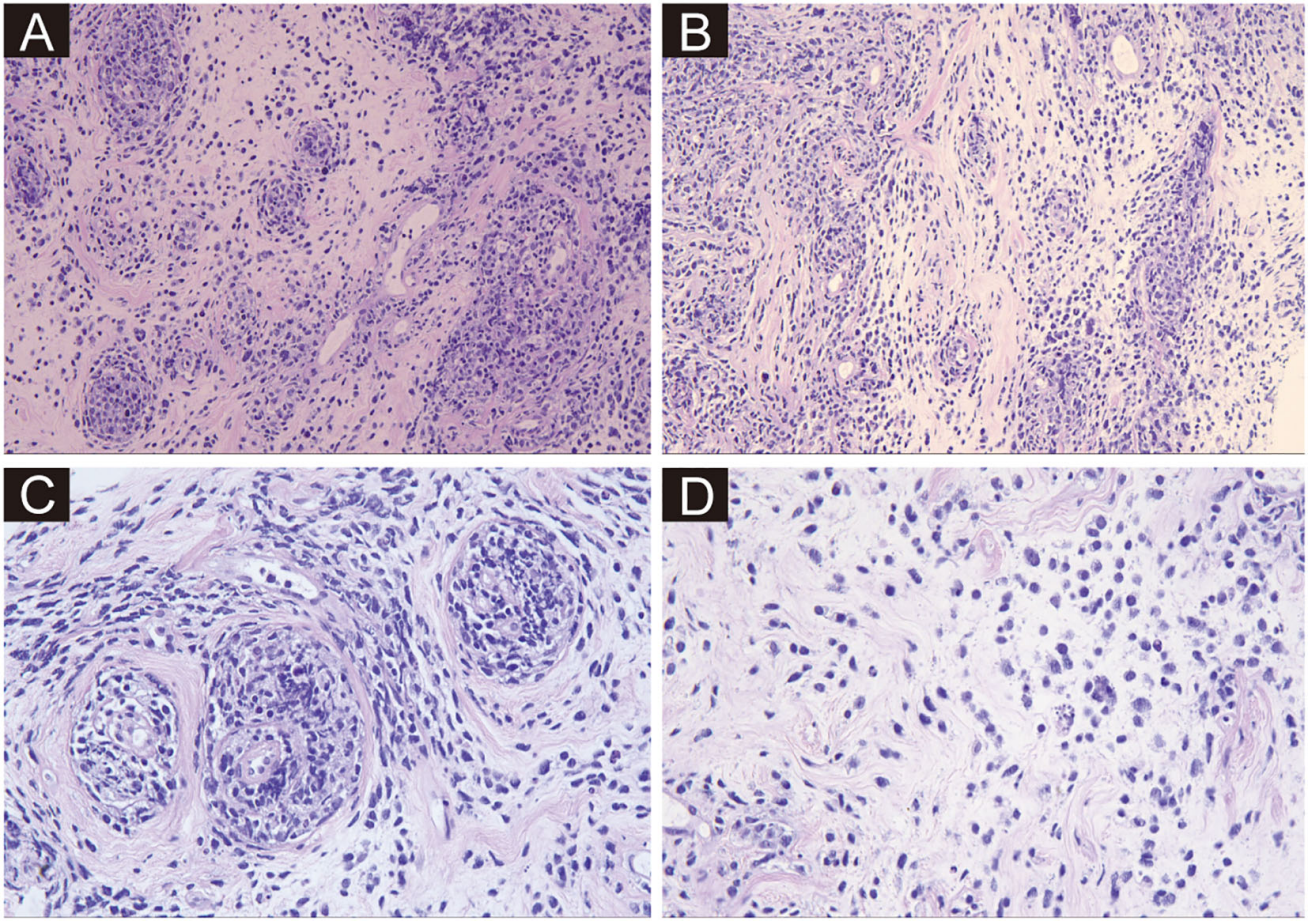
Hematoxylin and eosin-stained sections from needle biopsy. **(A–D)** Depict the neoplastic tissue at ×200, ×200, ×400, and ×400 magnifications, respectively. Atypical cells are arranged in small clusters, forming a target-like pattern around blood vessels, or distributed in a soldier-like or scattered pattern within the stromal tissue. The cells exhibit large cell size, irregular nuclear shape, coarse granular chromatin, and frequent mitotic figures.

A 34-year-old female presented to the local surgical department with complaints of right breast swelling and dull pain of unknown etiology for ten days. She denied a history of skin swelling, itching, ulceration or fever. Initial breast ultrasonography revealed thickening of the right breast parenchymal layer, with indistinct internal stratification and disorganized arrangement, measuring 6.9 × 2.3 cm. No enlarged lymph nodes were found. A proliferative lesion was considered. Meanwhile, the routine blood biochemical test showed normal leukocyte count, erythrocyte count, hemoglobin level, and platelet count. However, lactate dehydrogenase (LDH) was elevated to 365 U/L. The local surgeon considered that the patient was most likely suffering from a benign breast condition, attributing the mild LDH elevation to possible inflammation. Nevertheless, to exclude any potential for malignant neoplasms, the clinical team recommended a percutaneous biopsy for histopathological confirmation. The patient declined this suggestion and opted for a more conservative treatment approach. After 6 weeks of physical therapy, including hot compress and acupuncture treatment, the symptoms did not improve, and the swelling worsened, and she developed discomfort with a sensation of pressure during sleep. The patient was transferred to our hospital for further consultation. A repeat breast ultrasound, performed 2 months after the initial examination, was conducted at our hospital. On examination, a Large solid mass (13 × 12 × 4.7 cm) with ipsilateral axillary lymphadenopathy (3.1 × 1.5 cm) was noted in the right breast. Given the rapid enlargement of the mass and ipsilateral lymphadenopathy, the clinician highly suspected malignant breast tumor. Thus, a needle biopsy was promptly performed. In the breast biopsy specimen, atypical cells infiltration was observed, with some cells arranged in small clusters, others forming a target-like pattern around blood vessels, and still others distributed in a soldier-like or scattered pattern within the stromal tissue, resembling the growth pattern of lobular carcinoma of the breast (**Figure 1**). The cells exhibited large cell size, irregular nuclear shape, coarse granular chromatin, and frequent mitotic figures.

**Figure 2:**
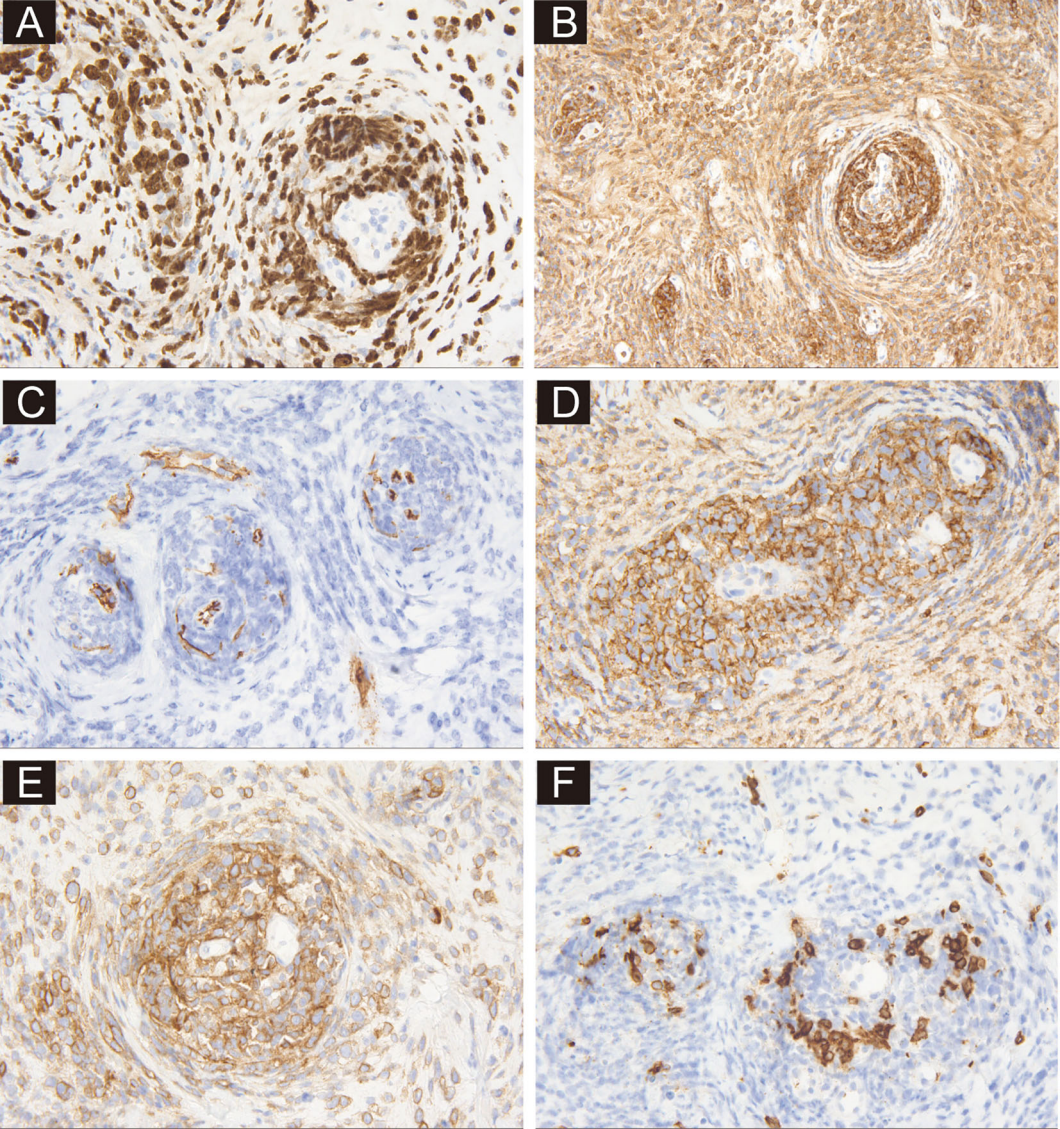
Immunohistochemical profiling of sections from needle biopsy. **(A)** Shows the immunoreactivity of tumor cells to Ki-67 at ×400 magnification. **(B)** demonstrates positive staining for LCA at ×200 magnification. **(C)** Shows positive staining for CD34 in vessels at ×400 magnification. **(D, E)** Demonstrate positive staining for CD20 and CD19 at ×400 magnification. **(F)** Shows the negative staining of CD3 at ×400 magnification.

The regular invasive breast cancer immunohistochemical (IHC) staining panel showed negative results for ER, PR, CK5/6, and E-Cadherin, while the Ki-67 index was 80% (**Figure 2A**). A malignant tumor was suspected, but the immunophenotyping was clearly inconsistent with breast cancer. After re-examination of the slides, the unique histological growth pattern puzzled us, especially the perivascular target-like growth pattern. Thus, the next round of IHC was performed to clarify the tumor origin. The tumor cells were strongly positive for LCA (Leukocyte Common Antigen), but negative for AE1/AE3, Desmin, and S-100 (**Figure 2B**), suspecting it could be a hematopoietic malignancy, including myeloid sarcoma, B-cell lymphoma and T/NK-cell lymphoma. A third round of IHC staining was performed, which showed negative expression for myeloperoxidase (MPO) and CD34—ruling out myeloid sarcoma and supporting a diagnosis of lymphoma (**Figure 2C**). It was positive staining for CD20, CD19, TdT, and negative for CD3, CD5, CD30, which helped us make a diagnose of diffuse large B-cell lymphoma (**Figures 2D–F**). Further classification showed positive for MUM1, Bcl2, BCL6, and negative for CD10 and C-MYC. EBER was negative by *in situ* hybridization, and *MYC* rearrangement was negative by FISH test. The diagnosis of DLBCL, NOS was made after three rounds of IHC.

**Figure 3:**
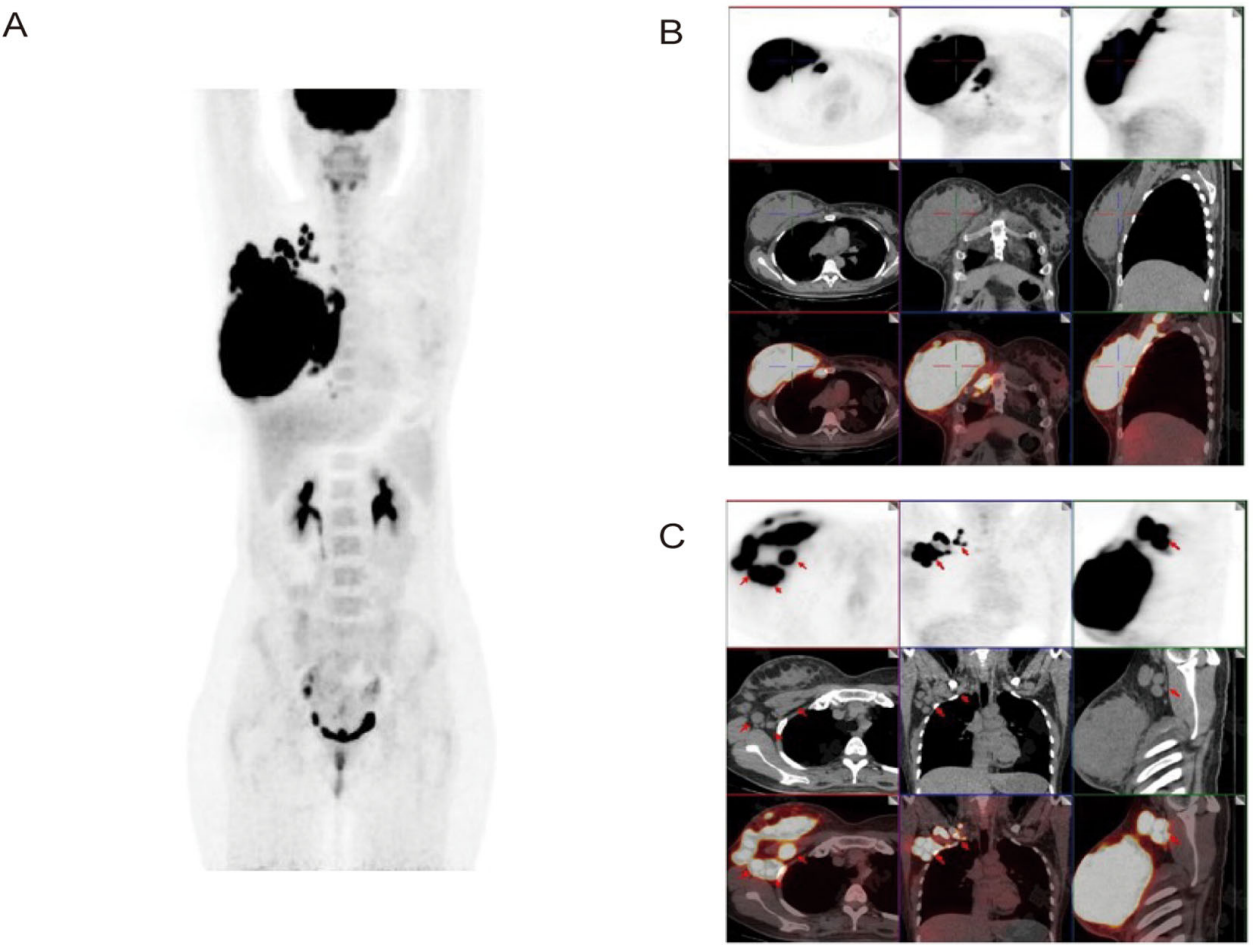
Axial and coronal reconstructions of PET scan (18F-FDG). **(A, B)** Demonstrate hypermetabolism of a large right breast mass (SUV 40.7) without evidence of distant adenopathy or secondary lesions. **(C)** Shows multiple enlarged lymph nodes in the right thoracic space and right axilla.

To further clarify the diagnosis and determine the stage of the disease, the clinician ordered additional examinations. Bone marrow biopsy specimen revealed no tumor cell infiltration, and a brain MRI scan did not reveal additional sites of disease, indicating no evidence of hematological and CNS involvement. PET/CT scan was also performed, providing a comprehensive evaluation of metabolic activity in the suspected areas. The scan revealed hypermetabolism of a large right breast mass (standardized uptake value (SUV 40.7)), and multiple enlarged lymph nodes in the right supraclavicular fossa, posterior sternum, right internal milk lymph chain, right thoracic space and right axilla, without evidence of distant adenopathy or secondary lesions (**Figure 3**). Therefore, the diagnosis of primary DLBCL of the breast was confirmed. Based on these imaging and clinical findings, the patient was staged as IIE according to the Ann Arbor staging, with an International Prognostic Index (IPI) score of one and a Central Nervous System International Prognostic Index (CNS-IPI) score of two.

**Figure 4:**
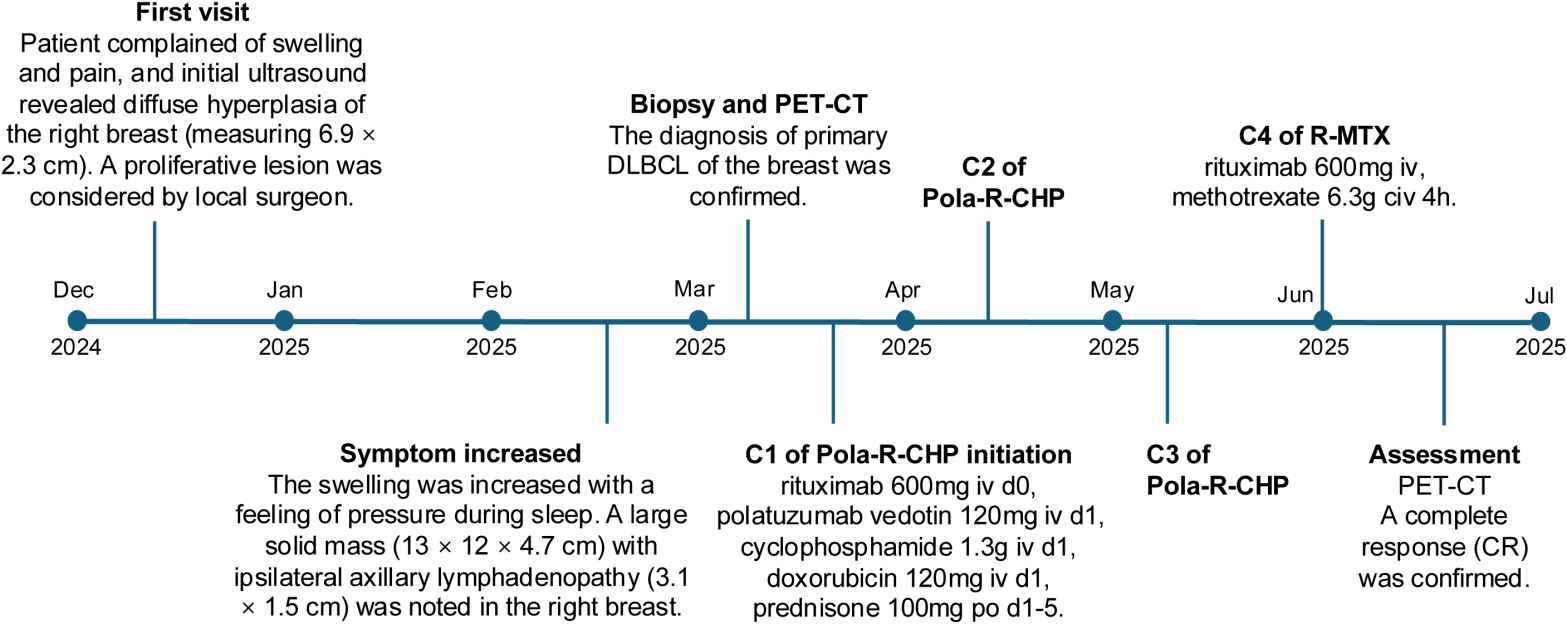
Timeline of the patient’s diagnosis and treatment.

The patient received three cycles of the Pola-R-CHP regimen (rituximab 600 mg intravenously [IV] on day 0, polatuzumab vedotin 120 mg IV on day 1, cyclophosphamide 1.3 g IV on day 1, doxorubicin 120 mg IV on day 1, and prednisone 100 mg orally [PO] on days 1–5) administered every 21 days, followed by one cycle of the R-MTX regimen (rituximab 600 mg IV and methotrexate 6.3 g continuous intravenous infusion [CIV] over 4 hours). After the 4 cycles of treatment, a repeat PET/CT was performed to assess treatment response, and a complete response (CR) was confirmed. The hypermetabolic foci in the original right breast, right cervical lymph nodes, and right thoracic lymph nodes have all shown a significant decrease in tracer uptake (Deauville score = 2) and there were no new lesions of increased metabolism. The patient demonstrated good compliance. No obvious adverse effects occurred during the treatment period. The patient was satisfied with the correct diagnosis and treatment and was optimistic about the prognosis. Considering the good effect of the treatment plan and economic reasons, the patient has been transferred to a local hospital for further treatment. Timeline of the patient’s diagnosis and treatment was shown in **Figure 4**.

## Discussion

As we know, breast cancer mostly arises from either the epithelial or stromal cell components of the breast parenchyma. In contrast, primary breast lymphoma (PBL), which arises from breast lymphoid tissue, is very rare. Primary breast DLBCL is even rarer with a prevalence less than 0.5% of all breast malignancies (9). Given the marked differences in treatment and prognosis, distinguishing primary breast DLBCL from breast carcinoma is critical. As a highly aggressive tumor, DLBCL requires systemic treatments, including chemotherapy, radiotherapy and immunotherapy. R-CHOP regimen is the most frequently administered treatment. Recently, the POLARIX trial, published in 2022, revealed Pola-R-CHP regimen showing better outcomes compared to R-CHOP regimen (10). As mentioned earlier, the treatment was used in our case and achieved a satisfactory treatment response.

Unfortunately, it is difficult to distinguish PBL and breast carcinoma through clinical systems and imaging examination. PBL typically presents as a unilateral, rapidly growing, painless breast mass and sometimes accompanied with enlarged axillary lymph nodes, which are also the features of breast invasive carcinoma. Systemic “B symptoms” including unexplained weight loss, night sweats and fever may occur but is rare (9). PBL can affect a broad age range, though most patients are diagnosed in their sixth decade of life (11). On ultrasound, PBL commonly appears as a hypoechoic mass with irregular shape, variable margins, and usually parallel orientation, but lack of distinguishing features from invasive carcinoma (8).

In the current case, the patient’s young age initially led clinicians to attribute her presenting symptoms to a benign condition. However, due to rapid progression of symptoms, malignancy was suspected, and biopsy was performed. Therefore, histopathological examination of biopsy samples is crucial for confirming the diagnosis. Typically, in DLBCL, the tissue architecture of lymph nodes or extranodal sites is partially or totally effaced by medium-sized to large lymphoid cells (with large cells defined as having a nucleus the same size or larger than a macrophage nucleus, or more than twice the size of a small lymphocyte nucleus) that are arranged in a diffuse or vaguely nodular pattern. In DLBCL of breast, the tumor tends to infiltrate between mammary ducts without destroying them (6). In addition, immunohistochemical testing is crucial for definitive confirmation. The distinctive growth pattern of tumor cells in our case was very rare and confused us about the histological origin of the tumor. T The perivascular “target-like” or “soldier-like” distribution of tumor cells within the stromal tissue closely mimicked the growth pattern of the more common invasive lobular carcinoma of the breast. The target-like pattern around blood vessels is uncommon in DLBCL. However, the case has brought us a warning that LCA may be a good choice when the histological pattern is ambiguous or confusing. Especially when the tumor cells are diffused or clustered, and at the same time do not completely resemble lobular carcinoma or non-specific invasive carcinoma, not only E-Cadherin but also LCA should be added into the IHC panel.

## Conclusion

In conclusion, the case of primary diffuse large B-cell lymphoma of the breast reveals a rare perivascular target-like growth pattern of tumor cells. It provides valuable insights for the differential diagnosis of breast tumors of epithelial versus lymphoid origin. Given these findings, we emphasize that hematological malignancies should be included in the differential diagnostic spectrum for breast lesions. Notably, when the histological pattern is ambiguous, leukocyte common antigen (LCA) immunostaining can serve as a useful marker to guide the diagnostic direction.

## Data Availability

The original contributions presented in the study are included in the article/supplementary material. Further inquiries can be directed to the corresponding author.
